# Characterization of hemizygous deletions in *Citrus *using array-Comparative Genomic Hybridization and microsynteny comparisons with the poplar genome

**DOI:** 10.1186/1471-2164-9-381

**Published:** 2008-08-09

**Authors:** Gabino Ríos, Miguel A Naranjo, Domingo J Iglesias, Omar Ruiz-Rivero, Marion Geraud, Antonio Usach, Manuel Talón

**Affiliations:** 1 Centro de Genómica, Instituto Valenciano de Investigaciones Agrarias, Carretera Moncada-Náquera km 4.5, 46113 Moncada (Valencia), Spain

## Abstract

**Background:**

Many fruit-tree species, including relevant *Citrus *spp varieties exhibit a reproductive biology that impairs breeding and strongly constrains genetic improvements. In citrus, juvenility increases the generation time while sexual sterility, inbreeding depression and self-incompatibility prevent the production of homozygous cultivars. Genomic technology may provide citrus researchers with a new set of tools to address these various restrictions. In this work, we report a valuable genomics-based protocol for the structural analysis of deletion mutations on an heterozygous background.

**Results:**

Two independent fast neutron mutants of self-incompatible clementine (*Citrus clementina *Hort. Ex Tan. cv. Clemenules) were the subject of the study. Both mutants, named 39B3 and 39E7, were expected to carry DNA deletions in hemizygous dosage. Array-based Comparative Genomic Hybridization (array-CGH) using a *Citrus *cDNA microarray allowed the identification of underrepresented genes in these two mutants. Subsequent comparison of citrus deleted genes with annotated plant genomes, especially poplar, made possible to predict the presence of a large deletion in 39B3 of about 700 kb and at least two deletions of approximately 100 and 500 kb in 39E7. The deletion in 39B3 was further characterized by PCR on available *Citrus *BACs, which helped us to build a partial physical map of the deletion. Among the deleted genes, *ClpC*-like gene coding for a putative subunit of a multifunctional chloroplastic protease involved in the regulation of chlorophyll *b *synthesis was directly related to the mutated phenotype since the mutant showed a reduced chlorophyll *a*/*b *ratio in green tissues.

**Conclusion:**

In this work, we report the use of array-CGH for the successful identification of genes included in a hemizygous deletion induced by fast neutron irradiation on *Citrus clementina*. The study of gene content and order into the 39B3 deletion also led to the unexpected conclusion that microsynteny and local gene colinearity in this species were higher with *Populus trichocarpa *than with the phylogenetically closer *Arabidopsis thaliana*. This work corroborates the potential of *Citrus *genomic resources to assist mutagenesis-based approaches for functional genetics, structural studies and comparative genomics, and hence to facilitate citrus variety improvement.

## Background

The rapid increase of world population, the field degradation by soil salinization and erosion, and the likely fluctuations in climate caused by global warming will pose new and known challenges to agriculture during this century [1]. Crop improvements required to cope with these challenges could be attained through agronomic advances, leading to a better use of fertilizers, protection agents or soil rescue, and exploitation of recent technologies for plant breeding. Despite the outstanding importance of genetics-based breeding applied to spontaneous mutations and conventional hybrids, molecular and genomic tools are expected to develop their great potential for crop improvement through functional genetics analysis, involving gene and function discovery and genome modification.

Citrus, some of the most important fruit crops worldwide, are perennial trees requiring a juvenility period of several years and frequently are parthenocarpic and sexually self-incompatible [2,3], which considerably impairs traditional breeding. Genomic technology, including methods to rapidly identify and manipulate genes of agricultural interest, holds promise of improvements that may be difficult through traditional approaches. In recent years, *Citrus *has been the target of several genomic developments including large EST collections [4-7], cDNA and oligonucleotide-based microarrays [4,8,9], BAC libraries and BAC end sequencing (BES) (to be published). However, functional studies, i.e. genetic transformation and the capability to perform reverse genetic analyses, are also considerably impaired. In citrus, high throughput transgenic programs such as the generation of RNA interference knockouts, activation tagging through enhancer elements, gene-trap T-DNA insertions, or transposon tagging systems have not been developed yet. Since no efficient tagging or insertional procedures are available in these species, other gene disruption methods including strategies based on genome-wide mutagenesis such as TILLING and fast neutron mutagenesis have been initiated. These approaches are non-transgenic and may have particular interest for the industry where the debate on genetically modified organisms has restricted application of these technologies to crop improvement. Both approaches, however, are of limited usefulness as strategies for reverse genetics because of the lack of knowledge on *Citrus *genomic sequence and the large amount of space required for the establishment of mutant populations. ECOTILLING on natural citrus variants and microarray-based detection of deletions in fast neutron citrus mutants are apparently very straightforward approaches. In this work we explore the potential of this last idea using two fast neutron *Citrus clementina *hemizygous mutants from the IVIA collection and a 20K cDNA citrus microarray.

Physical mutagenesis through fast neutron irradiation has been reported to cause variable genomic deletions ranging in size from few base pairs to 12 kb in *Arabidopsis thaliana *[10,11]. Several approaches have been used to characterize plant genomic deletions at the molecular level. These mostly include positional cloning [12], a method applicable to any kind of genetic lesion that, however, needs highly saturated genetic maps; PCR-based reverse genetics techniques [11,13], requiring a previous considerable knowledge of genomic sequence; and genomic subtraction procedures [14-16], which do not need sequence information but are strongly dependent on the gene dosage. Since very little is known about *Citrus *genome sequence and the *Citrus *induced deletions are in hemizygous gene dosage, an array-based procedure as the one employed for identifying homozygous gene deletions in *Arabidopsis *[17] seems more suitable for our purpose than those methods. Although the main application of microarrays is transcriptome profiling analysis, microarrays can also be used to study DNA variation. Oligonucleotide arrays are particularly suited for the detection of single nucleotide mismatches during hybridization, and hence for the discovery of novel DNA variants or the determination of known variants. The origin of this technique relies on a cytogenetic method described 25 years ago named "Comparative Genomic Hybridization" (CGH) that used differential DNA hybridization on chromosome spreads for visualization of deleted or amplified genomic regions in tumour tissues [18]. Subsequently, different laboratories mostly working on cancer research independently applied microarray technology to genomic DNA hybridization procedure, a technique consequently named array-CGH [19-23]. Array-CGH was successfully utilized to detect gene duplications in *Arabidopsis *and rice [24], and to validate aneuploidy analysis performed by quantitative fluorescent PCR in *Arabidopsis *[25]. Therefore, this method has proven to be suitable to study chromosomal imbalances in plants.

For the characterization of the deleted regions we also leaned on comparative genomics with other dicots since available physical citrus maps are not yet integrated with known genetic maps. Comparative genomics takes advantage of available information on gene content and order in genomic DNA from different species to infer phylogenetic relationships and formulate hypotheses on DNA evolutionary dynamics. Whole genomes are preferentially compared when available, but more often relatively short stretches of DNA or polymorphic markers are used.

The main objective of this work was to identify deleted genes on a heterozygous genetic *Citrus *background, provided by fast neutron generated mutants, through array-Comparative Genomic Hybridization. In addition, we also explored the possibility of using comparative genomics with annotated dicot genomes assisted by BAC end sequencing for the generation of partial physical maps of the deleted *Citrus *regions.

## Results and discussion

### Procedure for the characterization of hemizygous deletions in *Citrus*

**Figure 1:**
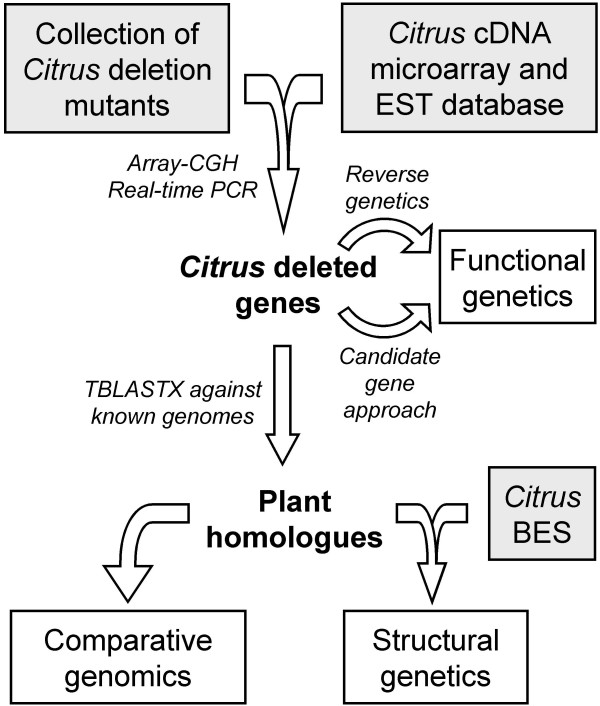
**Schematic guidelines for the characterization of hemizygous deletion *Citrus *mutants**. Arrows indicate successive steps of mutant characterization. Plant material and genomic resources are highlighted in grey boxes while gained knowledge of genetics and genomics are highlighted in white rectangles. Genes are shown in bold and methods and approaches in italics.

The proposed procedure to identify deleted genes is illustrated in Figure 1 and its potential to structurally characterize hemizygous deletions is exemplified below with *Citrus *mutants as starting plant material. Its usefulness to describe genomic deletions in other species might be dependent upon genome complexity and ploidy. This method uses cDNA microarrays to hybridize genomic DNA extracted from the deletion mutants to render a list of underrepresented genes. The putative deleted genes are then validated through gene dosage evaluation by real-time PCR using gene specific primers. Deleted genes could subsequently contribute to the identification of the molecular mechanisms underlying the observed phenotypes by means of a candidate gene approach, validated by physiological analyses or genetic transformation [26]. In non-sequenced genomes or in plants with poorly developed physical maps, further characterization of deletions at the structural level requires TBLASTX similarity searches against databases containing the sequence annotation of known eudicot genomes, such as *Arabidopsis thaliana*, *Populus trichocarpa *and *Vitis vinifera*. These searches yield putative orthologous genes and syntenic genomic regions between these four species. Local physical maps of deletions are built allocating the deleted gene sequences and the syntenic genomic fragments from these other eudicots into a BES database of the species of interest. Lastly, specific PCR on the array of BACs confirms gene content and order on the lineal structure of the deletions. The results may also be used in comparative genomics analyses to study evolutionary dynamics and phylogenetics.

### Identification of deleted alleles in 39B3 and 39E7 fast neutron mutants of *Citrus clementina*

For this study, two mutants obtained by fast neutron mutagenesis of wild type *Citrus clementina *were selected from the IVIA mutant collection. These mutants, named 39B3 and 39E7, were expected to carry DNA deletion lesions in hemizygous dosage and showed a delay in natural colour break in fruit peel. The 39B3 mutant exhibited a delay in colour change from green to orange while 39E7 was better characterized by an abnormal final yellowish colour instead of the natural orange coloration. Putative deleted genes in the mutants were first identified through an approach based on genomic hybridization (array-CGH) that exploited a recently developed *Citrus *microarray containing 21240 cDNAs [4,5]. To this end, total genomic DNA from four independent samples of mutants 39B3 and 39E7 were Cy3 or Cy5-labelled and cohybridized with wild type DNA labelled with the complementary Cy5 or Cy3 probe on four independent microarray slides. Fluorescence intensity data were normalized and single ESTs showing a mutant/wild type signal ratio lower than 0.7 fold, with a P-value lower than 0.2 (39B3) or 0.1 (39E7), were selected as putative candidates.

The number of ESTs fulfilling these criteria was 24 and 78 for mutants 39B3 and 39E7, respectively. One of the 39B3 positives [GenBank: CX299090], composed of three unrelated sequences was discarded for subsequent analysis due to its chimerical nature. In order to validate the array-CGH results, gene dosage of several putative candidates was determined through real-time PCR quantification of mutant/wild type signals for candidate ESTs as related to a reference undeleted gene [GenBank: CX293764]. The results showed that gene dosage for 39B3 candidates ranged from 0.50 to 0.60 when genomic DNA from the 39B3 genotype was tested, while ranged from 0.96 to 1.15 when the assayed DNA originated from the 39E7 genotype (Table 1). Similar results, corroborating the presence of putative deleted genes at half dosage, were also obtained for the 39E7 mutant. Therefore, the developed array-CGH procedure proved to be an appropriate tool to identify genes in hemizygous content in the self-incompatible clementine.

**Table 1:** Gene dosage measurement of deleted genes in 39B3 and 39E7 *Citrus *mutants.

Unigene	EST accession number (GenBank)	Array-CGH		Real-time PCR gene dosage	
		
		Mutant/wt ratio	P value	Template 39B3	Template 39E7
aCL4690Contig1	CX295702	0.59	0.10	0.56 ± 0.02	0.99 ± 0.09
aCL1915Contig2	DY300024	0.62	0.12	0.60 ± 0.08	0.96 ± 0.04
aCL3317Contig1	DY265056	0.62	0.10	0.60 ± 0.03	0.98 ± 0.03
aC20009H03SK_c	CX308429	0.63	0.10	0.50 ± 0.02	0.98 ± 0.11
aCL766Contig1	CX288964	0.65	0.13	0.56 ± 0.06	0.96 ± 0.11
aCL7097Contig1	FC868864	0.65	0.12	0.59 ± 0.04	1.15 ± 0.07

aCL2087Contig2	DY300006	0.57	0.05	1.05 ± 0.13	0.59 ± 0.11
aCL6684Contig1	DY265447	0.60	0.05	1.04 ± 0.12	0.59 ± 0.12
aCL6641Contig1	FC930062	0.62	0.05	1.14 ± 0.19	0.58 ± 0.08
aCL3902Contig1	DY267778	0.62	0.05	1.12 ± 0.10	0.64 ± 0.04
aC05139C12SK_c	CX296347	0.66	0.05	1.24 ± 0.22	0.61 ± 0.05
aC01019E12SK_c	CX288357	0.66	0.05	1.04 ± 0.07	0.63 ± 0.11

Accession numbers and the corresponding assembled unigenes for several candidate ESTs are shown. Array-CGH data are signal ratios of mutant with respect to wild type samples for each single EST. Gene dosage was calculated through real-time PCR on 39B3 and 39E7 genomic templates. A horizontal line separates array-CGH hits of 39B3 mutant (upper) from those of the 39E7 mutant (lower).

### Clustering of homologues of *Citrus *deleted genes in the poplar genome

**Figure 2:**
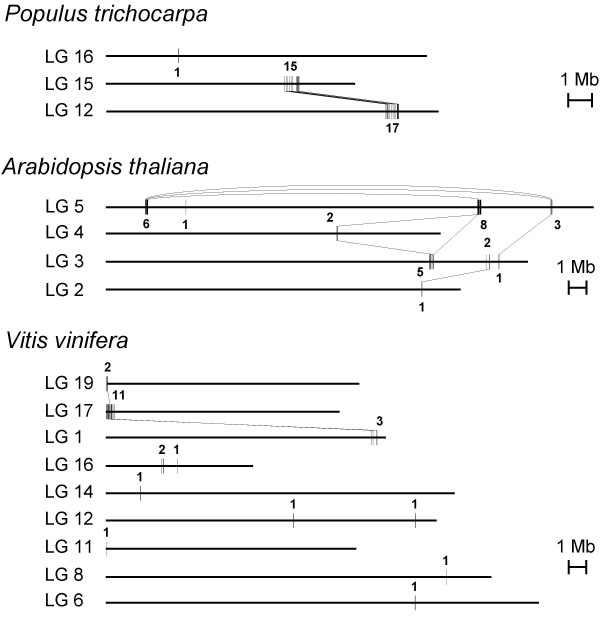
**Chromosome mapping of poplar, *Arabidopsis *and grapevine homologues of the 39B3 *Citrus *deleted genes**. The first TBLASTX hit for each *Citrus *deleted gene with an E value cut-off < 10^-5 ^is represented on linkage groups (LG) from *Populus trichocarpa*, *Arabidopsis thaliana *and *Vitis vinifera*. Homologues of *Citrus *genes were grouped into clusters in each species when the distance between them was shorter than 250 kb. Second and third hits are only represented when they are located in a previously identified cluster, and in this case are linked to the first hit by a line. The value on each cluster indicates the hit number of the cluster.

**Figure 3:**
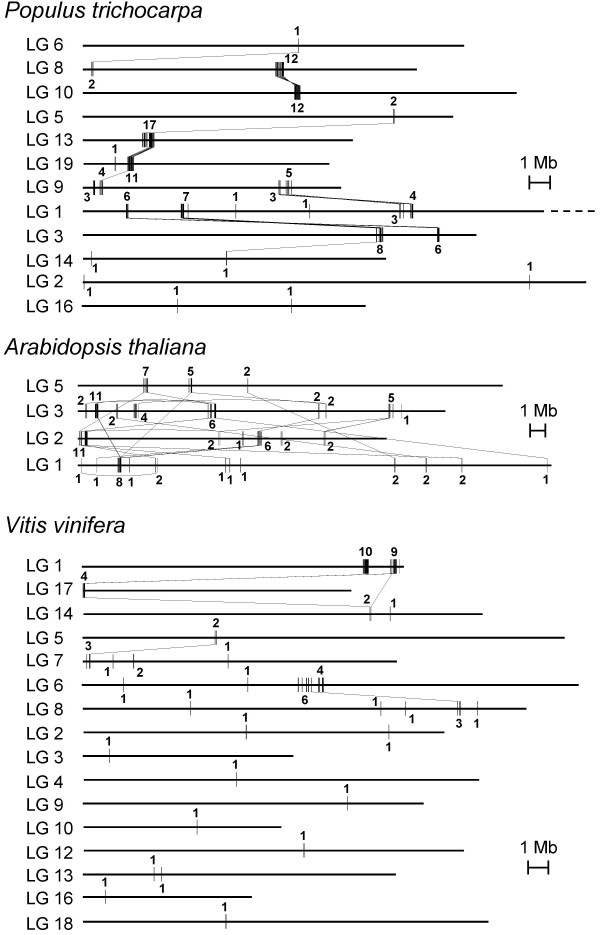
**Chromosome mapping of poplar, *Arabidopsis *and grapevine homologues of the 39E7 deleted genes**. The TBLASTX hits for the 78 39E7 candidates, identified as stated in figure 2, are represented on linkage groups (LG) from *Populus trichocarpa*, *Arabidopsis thaliana *and *Vitis vinifera*. The number of hits contained in each cluster is shown.

Microsynteny comparisons with homologous stretches from the sequenced genomes of *Arabidopsis thaliana*, *Populus trichocarpa *and *Vitis vinifera *[27-29] were performed in order to elucidate hypothetical clustering of *Citrus *deleted genes in the genome. TBLASTX, which searches for translations of a crude genome similar to a translated query, was utilized with an E-value cut-off of 10^-5^. The homologous regions produced by the best TBLASTX hit of each of the *Citrus *candidate genes were located on the chromosome maps of *Arabidopsis*, poplar and grapevine. Homologues of *Citrus *genes were then grouped into clusters in each species when the distance between them was shorter than 250 kb. The second and third TBLASTX best alignments were similarly placed in the respective maps when they were included in an existing cluster. In this case, a binding line was drawn linking the second and third hits to the best hit of the same *Citrus *query. Thus, two chromosomal maps, one for each mutant, in the three species was obtained. Figure 2 represents in detail chromosome mappings of the 39B3 mutation, which was subjected to further analyses. The results indicate that the *Populus *mapping exhibited rather lower complexity than the *Arabidopsis *and grapevine ones since it included fewer chromosomes and only 3 clusters although the number of 39B3 candidate genes represented in the map was identical (21) for the three genomes. Note that the number of represented hits in these mappings is higher than 21 due to the inclusion of second or third homologues. In *Populus*, most of the candidate genes mapped to two different genome regions of approximately 700 kb long in chromosomes 12 and 15, two duplicated chromosomes that probably originated during the recent genome duplication event that occurred in this species [28]. These two clusters contained 17 and 15 hits respectively while the third one placed in chromosome 16 had only one hit. In contrast, the number of clusters in *Arabidopsis *and *Vitis *were 9 and 11, respectively, and none of them contained more than 11 hits. Furthermore, cluster number (and clustering density) of the homologues of 39E7 putative deleted genes was also lower (and higher) in *Populus *than in *Arabidopsis *or *Vitis*, although the differences were smaller: 26, 30 and 30 clusters were obtained for poplar, *Arabidopsis *and grapevine respectively (Figure 3).

Overall, these observations suggest that the *Populus *genomic regions homologous to the *Citrus *deletions were less fragmented than their counterparts in *Arabidopsis *and *Vitis*, and consequently microsynteny on the considered segments was higher with the *Populus *genome. These results are striking since *Citrus *and *Arabidopsis *belong to Sapindales and Brassicales orders (inside the same clade eurosids II) while *Populus *is included in the eurosids I clade, and *Vitis *is part of Vitaceae, a family outside of rosids [30].

### Gene arrangement and partial physical map of the 39B3 deletion

**Figure 4:**
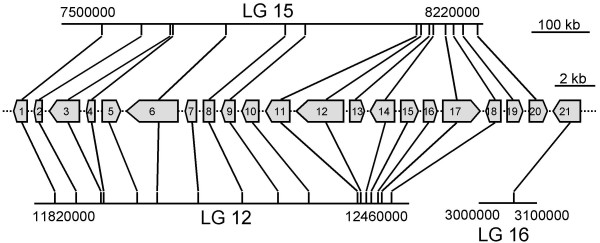
**Gene composition of the *Citrus *39B3 deletion inferred from poplar homologous regions**. The 39B3 deleted *Citrus *genes are arranged in the centre of the figure in the order inferred from the position of their *Populus *homologues found in linkage groups 12, 15 and 16. Genes are numbered following this order. Strand sense deduced from poplar counterparts is indicated by an arrow.

The closer microsynteny observed between the 39B3 deletion and the two duplicated homologous regions in poplar enabled prediction of gene order by direct inference from the *Populus *sequences. This assumption led to the gene arrangement depicted in Figure 4. Twenty genes out of twenty-one having high similarity with *Populus *homologues were directly located on the *Citrus *deletion fragment by combining the two clusters found on *Populus *chromosomes 12 and 15, which shared 12 hits. Inclusion of the 21^st ^gene, a homologue of a *Populus *gene placed on chromosome 16, in the 39B3 deletion was based on its location on the right end of the *Citrus *BAC CCER1019D04 (named B12, see below), whose left end shared identity with another deleted gene [GenBank: CX295702]. The accession number and protein similarity of these 21 genes, numbered according to the ordered position of their homologues on the poplar genome (Figure 4), are depicted in Table 2 that also shows coding strand sense of poplar homologues. The coding strand was coincident for the *Populus *paralogous genes present in chromosomes 12 and 15, except for genes similar to *Citrus *CX308429, located in position 8 in Figure 4.

**Table 2:** Gene components of the *Citrus *39B3 deletion.

N°	Citrus unigene	EST accession number (GenBank)	Strand	Similarity
1	aC01006D04SK_c	CX287243	-	Hypothetical protein
2	aC20006C06SK_c	CX308114	-	Ubiquitin conjugating enzyme
3	aCL3991Contig1	DY278065	-	Sterile alpha motif (SAM) domain-containing protein
4	aC18005F10Rv_c	CX305429	-	Sialyltransferase-like protein
5	aCL3317Contig1	DY265056	+	Hypothetical protein
6	aCL766Contig1	CX288964	-	ATP-dependent Clp protease, clpC homolog
7	aC01012C02SK_c	CX287682	-	Alpha-mannosidase
8	aC20009H03SK_c	CX308429	?	Mei2-like protein
9	aC16014F08SK_c	CX304691	-	Putative pol polyprotein
10	aCL6210Contig1	DY282423	-	Hypothetical protein
11	aCL8592Contig1	DY267639	-	Tudor domain-containing protein
12	aCL1065Contig1	DY282340	-	Putative amidase
13	aC32108G01EF_c	FC921733	+	Hypothetical protein
14	aCL503Contig1	CX292510	-	Respiratory burst oxidase homolog
15	aCL6269Contig1	DY263746	+	FHA domain-containing protein
16	aCL7097Contig1	FC868864	+	Putative pentatricopeptide (PPR) repeat protein
17	aCL4690Contig1	CX295702	+	ERD1 protein, chloroplast precursor
18	aCL8011Contig1	FC923875	-	Fe-superoxide dismutase
19	aIC0AAA60DF12RM1_c	DY284274	+	Poly(A)-binding protein II-like
20	aCL1848Contig1	FC875470	+	Hypothetical protein
21	aCL1915Contig2	DY300024	-	Tubulin-specific chaperone C-related

Unigenes are numbered according to the ordered position of their homologues on the poplar genome. The accession numbers correspond to 39B3 candidate ESTs. The coding frame of *Populus *homologues follow the proposed strand (+) or the complementary reverse one (-).

**Figure 5:**
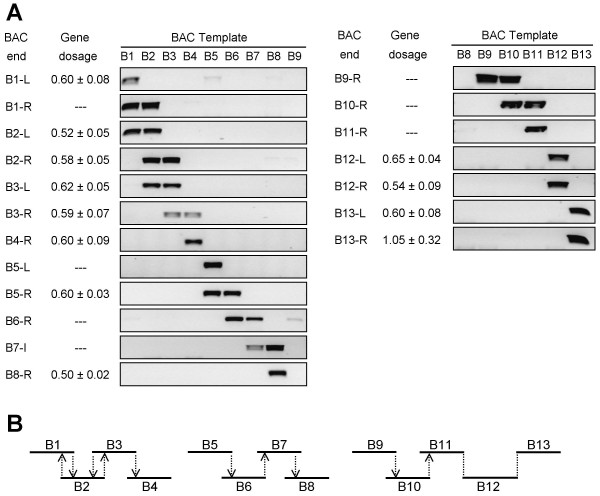
**Local physical mapping of the *Citrus *39B3 deletion**. (A) Electrophoretic analysis of PCR products showing overlapping BACs. Purified BAC templates are distributed horizontally and divided in two panels. Primer pairs were designed from BAC end sequences containing non-repetitive DNA and named with the number of the BAC plus "-L" for left end and "-R" for right end according to the drawing orientation. B7-I primers amplify an internal sequence from B7 instead of an end. Gene dosage measurements for some of the primer pairs in the 39B3 genotype are shown on the left side of the electrophoretic images. (B) Physical map of the *Citrus *39B3 deletion. Horizontal lines represent BACs, which are numbered from left to right. Vertical arrows show overlapping as inferred from PCR reactions and the head of the arrow indicates the BAC template. The vertical lines without arrow show connection of B11 with B12 by sequence of unigene aCL4690Contig1 and B12 with B13 by aCL1915Contig2 instead of a PCR reaction.

Furthermore, the recent sequencing of 46,000 *Citrus clementina *BAC ends (to be published) enabled the construction of a physical map of the 39B3 deleted region. To this end, two DNA sequences covering 700 kb along the *Populus *chromosomes 12 and 15, containing the genes homologous to the *Citrus *deleted candidates, were BLASTed against the *Citrus *BAC end database. The homology search identified 33 BACs with a BLASTN E value lower than 10^-5 ^for both paralogous regions. In subsequent analyses, redundant BACs were discarded, while additional candidate BACs were obtained by comparing these previous ones with the BES database to yield overlapping BACs. Moreover, BACs with both ends showing similarity to repetitive DNA that may cause ambiguous positioning and inaccurate gene dosage measurement were also discarded. Finally, a partial physical map containing 13 BACs systematically named B1 to B13 (Table 3) was provided by standard PCR of BAC end amplicons against BAC templates and *in silico *search of overlapping antiparallel ends (Figures 5A, B).

**Table 3:** Listing of BACs included in the *Citrus *39B3 deletion.

N°	BAC	Ends	BES ID (GenBank)	BLASTX against plant proteins	E value
B1	CCL021E18	B1-L	ET070583	Nhf	--
		B1-R	ET070584	gi| 91805627| hypothetical protein	9e-24
B2	CCL011O24	B2-L	ET086992	Nhf	--
		B2-R	ET086991	gi| 7576215| hypothetical protein	7e-47
B3	CCER1037B12	B3-L	ET077105	gi| 7576215| hypothetical protein	3e-90
		B3-R	ET077106	Nhf	--
B4	CCER1032N17	B4-L	ET101817	gi| 25411577| probable retroelement pol polyprotein	2e-06
		B4-R	ET101816	Nhf	--
B5	CCL011N15	B5-L	ET087145	gi| 6469119| mitochondrial phosphate transporter	5e-56
		B5-R	ET087144	Nhf	--
B6	CCER1045A09	B6-L	ET077286	gi| 92895029| Polynucleotidyl transferase (retrotransposon protein)	8e-63
		B6-R	ET077285	gi| 30027167| auxin response factor-like protein	6e-85
B7	CCH3037D01	B7-L	ET112059	gi| 87240692| Helix-loop-helix DNA-binding	1e-21
B8	CCER1005N09	B8-L	ET079746	Nhf	--
		B8-R	ET079745	gi| 79331867| AML1; RNA binding/nucleic acid binding	2e-09
B9	CCH3005L04	B9-L	ET081228	gi| 33113977| putative copia-type pol polyprotein	2e-85
		B9-R	ET081227	gi| 51968598| peroxisomal Ca-dependent solute carrier-like protein	2e-21
B10	CCER1033B14	B10-L	ET102435	gi| 51968598| peroxisomal Ca-dependent solute carrier-like protein	2e-37
		B10-R	ET102434	Nhf	--
B11	CCL011K21	B11-L	ET086761	gi| 25402907| protein F5M15.26 (retrotransposon protein)	4e-78
		B11-R	ET086760	gi| 14334878| putative ATP-dependent Clp protease ClpD	4e-57
B12	CCER1019D04	B12-L	ET098996	gi| 14334878| putative ATP-dependent Clp protease ClpD	4e-35
		B12-R	ET098995	gi| 6729532| putative protein	3e-28
B13	CCL032E17	B13-L	ET094320	gi| 6729532| putative protein	9e-34
		B13-R	ET094321	Nhf	--

BACs are numbered according to the ordered position in the deletion from B1 to B13. BES are named with the number of the BAC plus "-L" for left end and "-R" for right end according to the drawing orientation in Figure 5b. Nhf: no hits found.

This mapping contained three gaps, one at the 5' deletion junction and two internal ones (Figure 5B) delimiting three main BAC clusters, composed of B1 to B4, B5 to B8, and B9 to B13. BACs B11 and B12 were connected by unigene aCL4690Contig1 coding for a putative subunit ClpD of an ATP-dependent Clp protease, whose sequence was shared by both BACs. Similarly B12 and B13 interaction is mediated by unigene aCL1915Contig2 (Table 2, 3). Real-time PCR quantification of gene dosage for some of the BAC ends (Figure 5A) confirmed the presence of these sequences at half dosage in the mutant genotype, indicating that the 39B3 mutation is a hemizygous deletion. Indeed, all analyzed BACs covered an internal segment of the deletion except B13 that exhibited haploid gene dosage on the left end and diploid dosage on the right one, suggesting that B13 contained the 3' border of the 39B3 deletion.

The above results indicated that the microsynteny between *Citrus *and *Populus *genomes was high enough to predict gene arrangement and to build a partial physical map of a *Citrus *genomic segment of about 700 kb, as inferred from the length of poplar homologous regions. Nevertheless, the observation that a 700 kb *Citrus *fragment only contains 21 genes may result striking considering an average distance of 10 Kb between adjacent genes, as deduced from the estimations of *Citrus *genome size (367 Mb) and gene number (35,000–40,000). It should be noted, however, that the microarray used in these analyses contains between approximately 2/3 and 1/2 of the estimated gene content of the *Citrus *genome, which may account for a major part of the hypothetical "loss" of deleted candidates. While this is a weakness of the currently available Citrus arrays, non-attributable to the array-CGH procedure, more complete results are expected after the development of a more representative cDNA microarray. Other limitations of the method may be related to the differential hybridization potential of different cDNAs, including for instance cross-hybridizations. In this regard, oligonucleotide arrays are particularly suited for the detection of dissimilar DNA variants. Alternatively, synteny might be limited to several genes located on a bulk of non-conserved sequences inside this 700 Kb region, a possibility that may only be corroborated after genome sequencing.

Overall, the data indicated that the *Populus *genome is a useful model for comparative genomics which may be used to characterize hemizygous deletions in *Citrus*.

### The *Citrus *39B3 deletion shows higher local gene colinearity with *Populus *than with *Arabidopsis*

**Figure 6:**
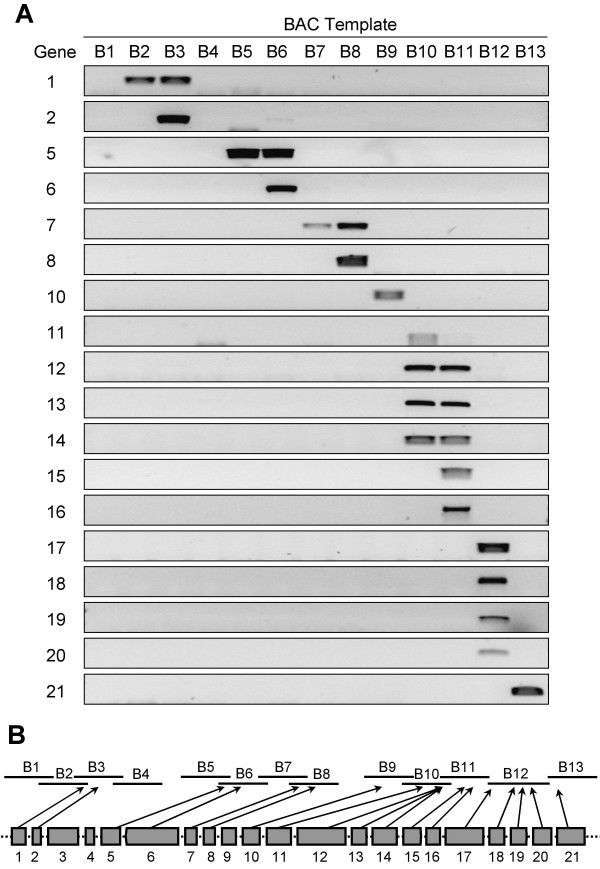
**Gene arrangement on the physical map of the *Citrus *39B3 deletion**. (A) Primer pairs designed for the putative deleted genes included in the 39B3 deletion (Table 2) were utilized in PCR reactions on the BAC templates shown in Table 3. Genes are numbered and arranged vertically, on the left side of the electrophoretic image, and BAC templates are listed horizontally. (B) *Citrus *genes included in the 39B3 deletion and arranged as drawn in Figure 4 but without indication of strand sense, are connected with arrows to the deletion physical map according to PCR results.

Local gene colinearity between two genomic fragments is determined by the number of paralogous genes arranged in the same order. Therefore, not only permanence of genes in their original chromosomal location, but also conservation of gene order, affects local colinearity. In order to validate the gene arrangement postulated in Figure 4 and consequently to estimate gene colinearity of the 39B3 *Citrus *deletion with *Populus *homologous fragments, we mapped by PCR the 21 genes listed in Table 2 on the physical map of Figure 5B. All but three genes showed at least one PCR product on the array of 13 BACs, confirming that those genes were effectively included in the 39B3 deletion (Figure 6A). In addition, PCR reactions reproduced at the BAC size resolution the expected gene order outlined in Figure 4, corroborating the gene arrangement deduced by comparative genomics. In Figure 6B, the genes rendering a positive PCR signal were linked to the physical map position with an arrow. Moreover, genes 3, 4 and 9 corresponding to unigenes aCL3991Contig1, aC18005F10Rv_c and aC16014F08SK_c, respectively, did not show a detectable PCR signal on purified BACs, although their respective primers produced a band of the expected size when tested against genomic DNA from normal clementine cultivar (data not shown). These genes were most likely placed into the two reported internal gaps of the physical map, as suggested by the border situation of their neighbouring genes.

These results confirm high local gene colinearity with poplar in the genomic region covered by 39B3 deletion. Taking together gene content and order conservation (Figures 2, 3 and 6), it is inferred that in the studied DNA deleted segment there was higher gene colinearity with *Populus*, which diverged about 109 million years ago (Mya), than with *Arabidopsis*, splitting from the *Citrus *lineage about 87 Mya [30], despite gene colinearity generally being correlated with phylogenetic relatedness. A similar conclusion has been reached in our group, after comparing the whole collection of *Citrus *BES with the poplar and *Arabidopsis *genomes (to be published), and also in previous works in papaya and melon. In papaya, BES alignment to the annotated genomes rendered higher gene colinearity with *Populus *than with *Arabidopsis*, although both *Arabidopsis *and papaya belong to the order Brassicales [31]. In melon, microsynteny studies based on the sequence of two BACs also concluded that melon was closer to *Populus *than to *Arabidopsis *or *Medicago truncatula *[32]. These observations may be explained by a differential genome evolutionary dynamics in poplar and *Arabidopsis *lineages [33]. The more recent appraisals estimated that last whole genome duplications occurred not later than 60–65 Mya in *Populus *and around 24–40 Mya in *Arabidopsis *lineages [28,34-36]. Despite the older poplar event, genome rearrangements involving gene loss and translocation following these duplications were much more frequent in *Arabidopsis *ancestors [37]. Such a highly active genome dynamics probably caused the dispersion of genes and the subsequent reduction in synteny and gene colinearity with even related species. The different behaviour of *Populus *and *Arabidopsis *ancestral genomes still deserves further explanation. It has been suggested that woody long-lived species like poplar trees may undergo a slower genome dynamics due to their juvenile period that delays sexual fecundation for several years and to the recurrent contribution of gametes from aged individuals of previous generations [28]. In addition, species like *Arabidopsis thaliana *may have very active mechanisms for unequal or illegitimate recombination causing frequent chromosomal rearrangements such as translocations, insertions and deletions. In this context, it is notable that nearly all *Citrus *species and many related genera have 2n = 18, probably indicating slow chromosomal evolution in this group.

### Chlorophyll *a/b *ratio is modified in 39B3 mutant

**Figure 7:**
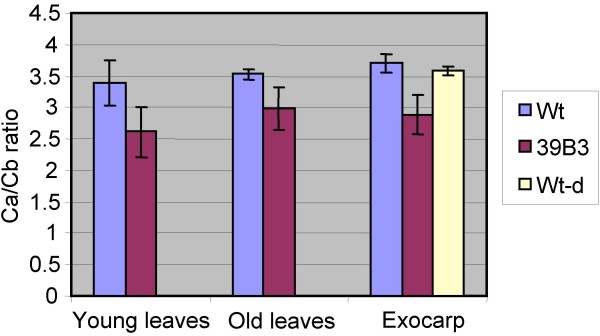
**Chlorophyll *a*/*b *ratio in green tissues from 39B3 mutant and wild type cultivar of *Citrus clementina***. Chlorophyll *a *and *b *content was measured in young and old leaves and fruit peel exocarp from wild type clementine cultivar (Wt) and 39B3 mutant (39B3). Measurements were also taken from exocarps of a normal wild type clementine tree showing fruit colour delay (Wt-d) due to altered environmental conditions. The relative content of chlorophyll *a *to chlorophyll *b *is represented as the Ca/Cb ratio. Data are average of 3 (exocarp) or 5 (leaves) independent determinations and error bars show standard deviation.

Structural studies describing gene arrangement on a particular deletion have outstanding importance for linking a specific mutant phenotype with an impaired gene. The 39B3 deletion removed at least a set of 21 genes and resulted in delayed chlorophyll catabolism. Although in principle, no obvious candidate genes could be unequivocally related to the exocarp colour break retardation, the 39B3 mutant certainly exhibited altered chlorophyll *a *and *b *content. Ratios of chlorophyll *a *to chlorophyll *b *contents in 39B3 mutant were about 15% to 23% lower than those found in wild type when three different green tissues were tested: fruit exocarp, old and young leaves (Figure 7). This distinct chlorophyll composition was not accompanied by alterations in the total content of chlorophylls in the leaves although pigment levels in 39B3 fruit exocarp, as expected, were clearly higher (0.48 mg/g fresh weight) than in the peel of control fruit (0.15 mg/g fresh weight) that has initiated chlorophyll degradation (Table 4). The chlorophyll accumulation observed in the 39B3 exocarp, however, is higher than the maximum reached in normal clementine fruits (0.35 mg/g f w) [38], suggesting that the mutation also induced total chlorophyll build-up in the fruit peel. Indeed, fruit exocarps of a "wild type" clementine tree showing fruit colour delay due to altered environmental conditions showed chlorophyll *a*/*b *ratios equivalent to those found in the standard cultivar (Figure 7) while total pigments had an intermediate value (0.29 mg/g f w) between those of normal and 39B3 genotypes (Table 4).

**Table 4:** Total chlorophyll content in green tissues from 39B3 mutant and "wild type" cultivar of *Citrus clementina*.

	Young leaves	Old leaves	Exocarp
Wt	0.76 ± 0.25	2.23 ± 0.19	0.15 ± 0.05
39B3	0.71 ± 0.24	2.49 ± 0.17	0.48 ± 0.01
Wt-d	--	--	0.29 ± 0.01

Total chlorophyll content (mg/g fresh weight) was measured in young and old leaves and fruit peel exocarp from samples shown in Figure 7. Data are average of 3 (exocarp) or 5 (leaves) independent determinations. Standard deviation is shown.

**Figure 8:**
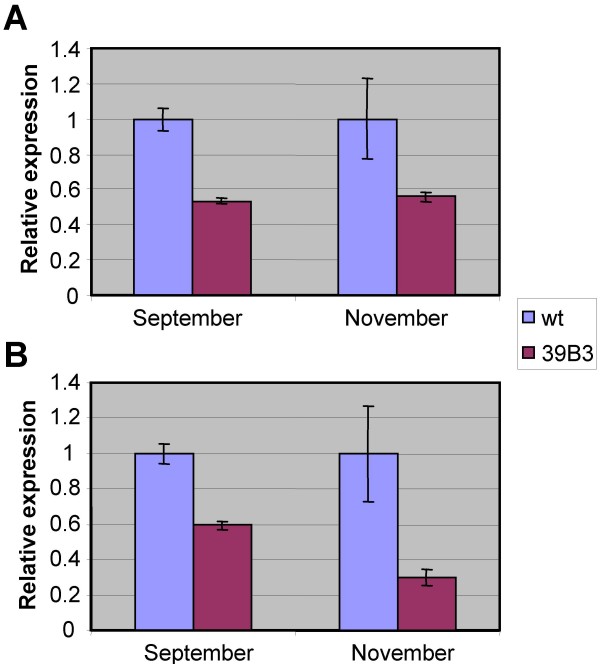
**Relative expression level of *ClpC*-like and *ClpD*-like genes in 39B3 mutant**. Quantitative real-time PCR with specific primers for *ClpC*-like (A) and *ClpD*-like genes (B) was performed on reverse transcribed RNA from fruit exocarps at two different developmental stages (September and November) from wild type (wt) and 39B3 mutant. Specific first strand cDNA concentration in 39B3 mutant is related to wild type values. The results are average and standard deviation of three independent biological replicates that were assayed twice.

**Figure 9:**
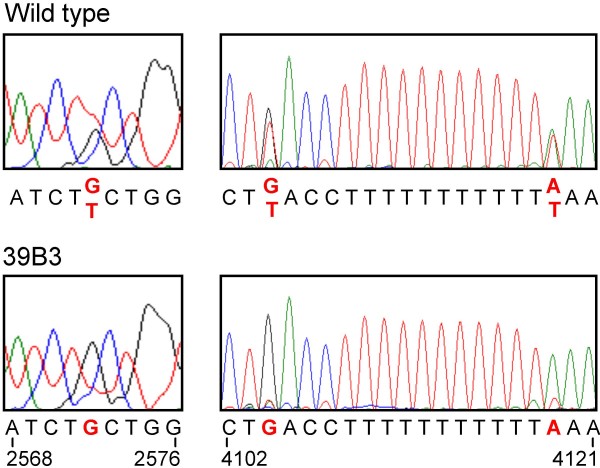
**Single nucleotide polymorphisms in *ClpC*-like alleles**. Four colour representations of polymorphic stretches in the sequence of the *ClpC*-like gene in wild type (upper panels) and 39B3 mutant DNA (lower panel). Differential nucleotides are labelled in red. The residues are numbered from the A in the ATG codon.

Unigene aCL766Contig1, one of the 39B3 hits validated by real-time quantitative PCR (Table 1) coding for a ClpC-like protein, may have certain relevance to the altered chlorophyll composition found in 39B3 mutant. Plant ClpCs are ATP-binding proteins located in the stroma of chloroplasts which have been found to be associated with the protein import machinery [39] and with the Clp protease complex [40]. In fact, ClpC has been related to protein translocation across the chloroplast inner envelope membrane and to multiple processes requiring proteolytic cleavage, as protein turnover and regulation [41,42]. In *Arabidopsis*, insertional mutagenesis in the *ClpC1 *gene caused chlorosis, growth retardation, photosynthetic damage and defects in chloroplast protein import [43-45] and no double knock-outs of *ClpC1 *and the less expressed *ClpC2 *genes were obtained, suggesting that *ClpC *function is essential in plants [46]. In addition, a mutant impaired in *ClpC1 *mRNA processing accumulated chlorophyllide *a *oxygenase protein (CAO), a key enzyme for the synthesis of chlorophyll *b *from chlorophyll *a*, leading to a reduced chlorophyll *a*/*b *ratio [47]. Interestingly, aCL4690Contig1 unigene coding for another subunit of Clp complexes (*ClpD*-like) with sequence similarity to aCL766Contig1 showed half gene dosage (Table 1) and was also included in the 39B3 deletion. Expression of both *ClpC*-like and *ClpD*-like genes was analyzed in fruit exocarps from wild type and 39B3 mutant at two different developmental stages: green immature peel (September) and shortly after the time of natural colour break in wild type peel (November). Both genes showed reduced expression in the 39B3 mutant, an observation that was well correlated with the alteration in chlorophyll composition since *ClpC*-like and *ClpD-*like alleles in the hemizygous 39B3 mutant reached about a half of the expression values found in the wild type (Figure 8). These results suggest that wild type alleles are similarly expressed in the peel of clementine fruit. Furthermore, sequencing of the *ClpC*-like gene that according to its *Arabidopsis *homologue plays a major role in chlorophyll composition, also revealed that there were no essential differences between wild type and 39B3 mutant coding regions (Additional file 1). This observation corroborated that not only expression but also protein sequence were identical in the analyzed *ClpC*-like alleles. In the wild type, three single nucleotide polymorphisms (SNP) were observed in two different introns, which were very likely unable to alter protein stability or function. These single base variants that were detected as sequence ambiguities (N) were due to the presence of overlapping base peaks contributed by both alleles, while hemizygous *ClpC*-like gene in 39B3 mutant produced an unambiguous signal in the same positions. Bases 2572, 4104 and 4119 located on the forth and sixth introns were identified as guanine, guanine and adenine in the mutant, while a mix of guanine and thymine, guanine and adenine and adenine and thymine were respectively found in wild type DNA (Figure 9). While the contribution of the *ClpC*-like gene dosage to the retardation of the natural exocarp degreening remains to be unequivocally demonstrated, the data presented above clearly shows that there is a strong correlation between chlorophyll composition and the presence of a single allele in the mutant. Additional analysis of the remaining genes inside 39B3 deletion should be performed in order to accomplish a complete candidate gene approach

## Conclusion

In this study, we propose a procedure for the genetic characterization of genomic hemizygous deletions in citrus mutants. The procedure that might be applied to other non-sequenced species of similar genome size and ploidy level is illustrated with the study of the 39B3 *Citrus clementina *deletion, generated by fast neutron bombardment. The proposed strategy utilizes several genomic resources such as array-Comparative Genomic Hybridization (array-CGH) technology, EST and BAC end sequencing databases and poplar genome annotation.

The array-CGH results led to the conclusion that the 39B3 deletion removed at least 21 genes while a partial physical map of about 700 kb of the deleted region was inferred by comparison of two homologous genomic regions from poplar with a *Citrus *BES database.

Structural data including gene content and order in the deletion was utilized for microsynteny and local gene colinearity studies concluding that in the studied region *Citrus *is more similar to *Populus *than to *Arabidopsis*, a phylogenetically closer species. This observation supports previous works on other species and suggests that the *Arabidopsis *lineage underwent a quicker genome evolutionary dynamics than the *Populus *one.

Among the deleted alleles, the function of *ClpC*-like, coding for a putative subunit of a protease involved in chlorophyll *b *synthesis was directly related to the mutant phenotype since green mutant tissues had a lower chlorophyll *a*/*b *ratio.

## Methods

### Plant material

Approximately 6 years-old clementine trees (*Citrus clementina *Hort. Ex Tan. cv. clemenules) grown under standard agricultural practices at the Instituto Valenciano de Investigaciones Agrarias (IVIA) were used in this study. Commercial highly heterozygous clementine cultivars are considered "wild type" material, while the 39B3 and 39E7 genotypes that belong to the IVIA mutant collection were obtained through bud irradiation with fast neutrons (5–6 Gy) at the Instituto Tecnologico e Nuclear (Sacavem, Portugal) in the frame of a much wider breeding program. Both mutants are expected to carry DNA deletion lesions in hemizygous dosage and showed altered patterns of colour change of fruit peel.

### Array-CGH

The protocol was adapted from several published array-Comparative Genomic Hybridization (array-CGH) methods pursuing mainly the measurement of copy-number changes in human genomic DNA [48-50], and the study of large-scale genetic variation of the symbiotic bacteria *Sinorhizobium meliloti *[51]. Genomic DNA was isolated from leaves of wild type and mutant plants, using DNeasy plant mini kit (Qiagen). Four Cy3 or Cy5-labelled independent biological samples from each mutant plant were co-profiled on four 20K *Citrus *cDNA microarrays containing 21240 EST, using Cy5 or Cy3-labelled control genomic DNA, respectively. Label probes were prepared as follow: Cy3- or Cy5-dCTP fluorescent nucleotides (Amersham Biosciences) were incorporated directly in control and mutant genomic DNA (2 μg) using BioPrime Array CGH Genomic Labelling System (Invitrogene). Purified Cy5 and Cy3 labelled probes (about 50 μl each) were combined and mixed with 30 μg Cot-1 DNA (Invitrogene), 100 μg yeast tRNA (Invitrogene), and 346 μl TE buffer pH 7.4. Cot-1 DNA and yeast tRNA were used to block non-specific hybridization. Samples were laid on a microcon YM-30 filter (Millipore), and subsequently centrifuged until sample volume was reduced to approximately 48 μl. Finally, 10.2 μl 20× SSC and 1.8 μl 10% SDS were added to the probe mixture to reach a final volume of 60 μl containing 3.4× SSC and 0.3% SDS. For microarray hybridization, the probe mixture was denatured by heating at 97°C for 5 minutes, and immediately incubated at 37°C during 30 minutes to block repetitive DNA sequences. Hybridization mixture was applied to a 37°C pre-warmed hybrid-slip (Sigma), and a pre-warmed array slide was lowered onto the mix. Microarrays were hybridized in darkness at 65°C overnight (16–20 hours) using a glass array cassette following manufacturer's instructions (Ambion, cat. n° AM10040). To prevent evaporation of hybridization solution during incubation, 5 μl of 3× SCC were poured into the reservoir inside the cassette chamber. Following hybridization, microarray slides were placed in a rack and the cover slip removed by 10 minutes immersion in a washing chamber containing 2× SSC and 0.03% SDS at room temperature (RT). Microarray slides were passed through a series of washes on a shaking platform. Wash series were as follow: 2× SSC, 0.03% SDS for 5 min at 65°C, followed by 1× SSC for 5 min at RT, and 3 × 15 min washes in 0.2× SSC at RT. After first wash slides were transferred to new racks to minimize transference of SDS to the next washing solution. Microarray slides were dried by centrifugation for 5 min at 300 rpm by using an Eppendorf 5804-R tabletop centrifuge. Arrays were immediately scanned at 5 μm. Cy3 and Cy5 fluorescence intensity was collected by using a ScanArray Gx (Perkin Elmer). The resulting images were overlaid and spots identified by the ScanArray Express program (Perkin Elmer). Spot quality was first measured by the signal-to-background method with parameters lower limit (200) and multiplier (2), and subsequently confirmed by visual test. Data analysis was performed using the Limma package from the R statistical computing software [52-54]. A mutant/wild type signal lower than 0.7, with a P-value not higher than 0.1 (39E7) or 0.2 (39B3) were the cut-off values for positive EST identification. The experimental design of microarray experiments has been loaded into the ArrayExpress database [55] under accessions E-MEXP-1432 and E-MEXP-1433.

### Gene dosage measurements

Quantitative real-time PCR was performed on a LightCycler 2.0 instrument (Roche), using the LightCycler FastStart DNA MasterPLUS SYBR Green I kit (Roche). Reaction composition and conditions followed manufacturer's instructions. Each individual PCR reaction contained 2 ng of genomic DNA from wild type or mutant, obtained with the DNeasy plant mini kit (Qiagen). Cycling protocol consisted of 10 min at 95°C for pre-incubation, then 40 cycles of 10 sec at 95°C for denaturation, 10 sec at 60°C for annealing and 10–25 sec at 72°C for extension. Fluorescent intensity data were acquired during the extension time. Specificity of the PCR reaction was assessed by the presence of a single peak in the dissociation curve after the amplification and through size estimation of the amplified product. For gene dosage measurements, we used the relative quantification-monocolor analysis from the LightCycler Software 4.0 package (Roche). This program compares the ratio of a target sequence to a reference DNA sequence in the mutant sample with the ratio of these sequences in a wild type sample. PCR and normalized calculations were repeated in at least three independent samples from each mutant and wild type, rendering an estimation of target gene dosage in the mutant genotype. Primers for the reference sequence were obtained from CX293764.

### Similarity searches

DNA sequences of *Citrus *unigenes containing positive array-CGH ESTs were used in online TBLASTX searches against genomic databases from the annotated genomes of *Arabidopsis thaliana *[56], *Populus trichocarpa *[57] and *Vitis vinifera *[58] at an E-value cut-off of 10^-5^. For each gene, the best hit was placed on a chromosomal map while the second and third hits were only positioned in the map if they were located closer than 250 kb to any other hit. Two 700 kb regions from chromosomes 12 and 15 from the *Populus *genome including homologous genes to 39B3 array-CGH positive unigenes, were used as queries in a BLASTN local search on a *Citrus *BAC end sequence database. Only hits corresponding to those BAC ends showing an E-value lower than 10^-5 ^in both chromosome searches were considered for the building of a local physical map of the 39B3 deletion.

### BAC isolation and analysis

DNA from *Citrus *BACs was isolated with the Rapid Plasmid Miniprep System (Marligen Biosciences). Purified BACs were used as templates in PCR reactions in a total volume of 15 μl, including 0.2 mM dNTP, 2 mM MgCl_2_, 0.5 μM of each primer, 0.38 units of Netzyme DNA polymerase (Molecular Netline Bioproducts) and 0.1 ng of BAC DNA. After an initial denaturing step for 5 min at 95°C, amplification was performed for 35 cycles of 30 sec at 95°C, 30 sec at 60°C and 30 sec at 72°C, followed by 5 min incubation at 72°C. The PCR product was subjected to 1.5% agarose DNA electrophoresis.

### Chlorophyll measurements

At least, three developing and mature leaves and fruit exocarp sectors from standard and 39B3 mutant lines of clementine were randomly collected per sample. Fruit exocarp tissues from a wild type clementine tree showing fruit colour delay due to altered environmental conditions were also sampled for chlorophyll analyses. Chlorophylls *a *and *b *were extracted with N,N-dimethylformamide for 72 h in the dark at 4°C and quantified through the absorbance at 647 and 664 nm following a reported procedure [59]. Absorbance was measured using a Varian Cary 50 UV-visible spectrophotometer (Varian).

### Gene expression measurements

Total RNA was extracted from fruit exocarp of wild type and 39B3 mutant using the RNeasy Plant Mini Kit (Qiagen). RNA concentration was determined by a fluorometric assay with the RiboGreen dye (Molecular Probes) following the manufacturer's instructions. About 5 μg of total RNA were reverse transcribed with the SuperScript III First-Strand Synthesis System for RT-PCR (Invitrogen) in a total volume of 20 μl. Single strand cDNA corresponding to *ClpC*-like and *ClpD*-like genes was amplified by quantitative real-time PCR on a LightCycler 2.0 instrument (Roche), using the LightCycler FastStart DNA MasterPLUS SYBR Green I kit (Roche). One μl of a 20 times diluted first-strand cDNA was used for each amplification reaction. Cycling protocol consisted of 10 min at 95°C for pre-incubation, then 40 cycles of 10 sec at 95°C for denaturation, 10 sec at 60°C for annealing and 15 sec at 72°C for extension. Melting curve analysis by applying increasing temperature from 65°C to 95°C (0.1°C/s) and gel electrophoresis of final product confirmed single amplicons. For expression measurements, we used the absolute quantification analysis from the LightCycler Software 4.0 package (Roche), and calculated expression levels relative to wild type values. Three independent biological samples were analyzed for wild type and mutant genotypes. Primers sequences are provided in Additional file 2.

### ClpC-like genomic sequence

*ClpC*-like genomic sequence from very few base pairs after the ATG until few base pairs before the stop codon was divided in four PCR fragments: Amplicon 3/4 (1820 bp) was amplified and sequenced with primers CLPC3 and CLPC4, amplicon 5/8 (2168 bp) was amplified with primers CLPC5 and CLPC8 and sequenced with primers CLPC5, CLPC8, CLPC10 and CLPC11, amplicon 7/2 (1446 bp) was amplified and sequenced with primers CLPC7 and CLPC2, and amplicon 1/6 (1158 bp) was amplified and sequenced with primers CLPC1 and CLPC6. Each amplicon was obtained by combining the product of 6–8 independent reactions. Primers sequences are provided in Additional file 2.

## Authors' contributions

GR carried out the microarray hybridizations, standard PCR reactions, expression measurements, gene sequencing, similarity searches and data analysis, and drafted the manuscript. MAN isolated DNA from *Citrus *BACs and carried out quantitative real-time PCR. DJI carried out mutant collection screenings, selected mutants and performed chlorophyll measurements. OR-R designed the array-CGH protocol. MG assisted in microarray hybridizations and data analysis. AU provided plant material, identified mutant genotypes and carried out relevant work on the field. MT conceived and coordinated the project and elaborated the final manuscript. All authors read and approved the final manuscript.

## Supplementary Material

Additional file 1 Nucleotide sequence of *ClpC*-like coding region plus introns in wild type and 39B3 mutant. Click here for file

Additional file 2 Primers employed in real-time and standard PCR experiments. Click here for file
