# Supplementary material for: Characterization of hemizygous deletions in Citrus using array-Comparative Genomic Hybridization and microsynteny comparisons with the poplar genome
Source: BMC Genomics. 2008 Aug 9;9:381. doi: 10.1186/1471-2164-9-381 (PMC2533677; doi:10.1186/1471-2164-9-381)
Supplement: Additional file 1 — Nucleotide sequence of ClpC-like coding region plus introns in wild type and 39B3 mutant. [file 1471-2164-9-381-S1.doc]

Genomic sequence of *ClpC*-like gene from start to stop codon (bold). Sequences deduced from database cDNAs are lowercase and the ones obtained in this work are uppercase. Introns are shown in red and polymorphisms in blue.

Wild type

**atg**gctaggcttctggctcagttaactaatgcccctgctttggttcctggcggaaGACATTTCCAGTCCCAAAATGCTGGAAGATCAAGAAGGAATGTCAAAATGATGTCTTATATGCAGCCGTCTGCTTCAAGAATTAGTAGTTTCTCAGGATTGCGACGCTCCAATGCTTTAGATACATTCACCTGTGATTTCCATTCCACGGTGGCTGTTTCACTCTCTTCTAGACGAGGAAAGTCTGGAAGAGCTGGTGGATTTGTTGTTAAAGCCATGTTTGAGCGCTTCACAGAGAAAGCAATTAAAGTGATTATGCTTGCCCAAGAGGAAGCAAGACGGCTTGGCCACAATTTTGTTGGCACGGAGCAAATTCTATTGGGTCTTATTGGTGAAGGCACTGGCATTGCTGCTAAGGTTCTTAAATCAATGGGAATCAATCTTAAAGATGCACGTGTAGAAGTGGAGAAGATTATTGGAAGGGGAAGTGGTTTTGTTGCTGTGGAAATTCCGTTCACTCCTCGTGCAAAGCGTGTCTTGGAGCTTTCTCTGGAGGAAGCCCGCCAGCTGGGTATCATAACCTTCTCCTTTCCAGTTTAGCAATGCCTTATGTTTTAGGAACTAGGATCTTTTTGGATTTCACTATAGGATTACTGTTTTAAATTCTGTTGGCCTGTCTCTTAACTGCGTGATTGAGCGTATCAGGTGCTTTAATTTTTGATATATTATCCGTTGGAACCTTTGCTTCGGGTCATTTGTTATTGGCCATATTTTGGGCTTAATCCTTCTACATATGTATCACTTTGATTTCCAAGTTCTTGAAGCCAAATTCTCTCAAATTTCAATCTCAGAAACTCACAAAAGTTTATATATGTGATGAAATGTTAAAAAATCATAGAGAAAAGTTGTTTAAGTTGATTCTTTAACCTTGAATTAATTATCTAATTTGTTATTGACTTTATTAGATCTAGAATTGGTTTTCCATTTGATTCTACTGAACTAAAGAAAATATAAATTAAAGCAATTTTTTTTTTGTTACATTGTTACTTTTGTAGCATCCAATTCATATGACCTTTGTTTCTGATTTTTAAAAGCAAGTGTTAGCTTCTTGAAATATATTATTTTCATTTTCTGACTAGGAAACACATCTAATTGTCATCTGAAAAATGTATACTTACTTGAAATTTCAGGCCATAATTATATTGGGTCCGAGCATCTTCTCCTTGGGCTTCTTCGTGAAGGGGAAGGTGTAGCAGCTCGTGTTCTTGAGAATTTAGGTGCTGACCCTAGTAACATCCGCACACAGGCAAGCAACAGTTCTTATCATTTGTGTTCCGATCTGATTTTCTTTCCTTGAGACCTGTTTCATTGCTAGCTTTGTTAAATAAAAAATTGATTTGATATTCTTAGTAAAAGATATAAATAGGCAAAGCTTTTGTTGGAGTGAACAGGTTATTCGTATGGTTGGTGAGAGCACAGAAGCTGTTGGCGCTGGTGTTGGAGGAGGAAGCAGTGGCAACAAGATGCCAACACTGGAGGAGTATGGAACAAATTTGACTAAGCTAGCAGAGGAGGTAATATGCGGTGACGTACATTGCAAAATTTGCTACTTAGTTTTTGAGGCATTTTTCTAAGTTTAGTTTCTTCTCGTTTCAGGGAAAATTGGATCCTGTTGTTGGGAGACAGCCACAAATAGAGCGTGTTGTCCAAATTTTGGGCCGTCGAACTAAGAATAATCCATGTCTTATTGGAGAACCTGGTGTGGGTAAAACAGCAATTGCAGAAGGCCTTGCTCAACGGATTGCAAGTGGTGATGTTCCTGATACCATTGAAGGAAAGAAGGTTGGTATAATCAGATATAGTTGTTAGCTATTTAACATGGGTACATTAACATTTTGAATCTTATAATCGAGGATGAAAGAACTTCCTTACCTCGTTTTTGAAGAATTTAATAGGAGCATTTGCCAACTTTGAGCTGTACTGGTCTGTATGTATATATGTGAACTCTTTGCATTAAAAAAGTTGCTTTTTCTTTTTTTGCATTTTTTTAACTAATTGAAAGTTATACCATAAGCTCTCTCTCTGAGATTTAAAGATGGCTGGTTAAGTGTCATTCAGAAGCTTTTGAATAATAGTCAGTCGTCTACTTGACATGGATACTTCATTTATTCCGAAGGCCAATCTTTCATATGACAGCTTGTGGATCTGGTGATGGGAATTTGATTTTTTTTTGTCATTTAAAATTTTGCATTTATTACTTGGAAAAATGTGTATCGTTAGTGTGAATTAAAAAAAAAAAACTGTTCGTATCATAAATGAATATATAATTAGGCATGTTGCACAAGAAGATGATATGAAGCATTTTATTTCAACTTCAATTCTGTTTGCAGCTTTACATATTTATGTAGTTATATTGATTTGGTGTGTGAACATTTAAATGATATGAAAAACCTTTTGAGTTAATTTAGATGTGTACTCATGTCATGTAGATGTTTTTTCTGCACAATATATTTGTCGATGGATTCGACTCTTGTTCTAAATATTTGATATTCATTTTGAAGAGAGAATACTTTATCTNCTGGTGATTGCTTGATTTGTATTCAAATGCACCAGAGTTATTGGATGAATGTAACCATGTGTATCCTGTAAAAAAGATGTAAGATTTCTGCTGCAAAACTTTAAACTGGCCTTTGACGCTTCTTCATTAGGTGCAGCGTGAATTTATAATTTCTCATAGAATGCTGTAACCTGAGGCTAGGTGGTTGCCACTAAATGAAGGATGTGGTGACCATTTATCATAATTTTATGTCCTGAAATTCAAATTTTTACACTGTTTGATGCTCTGGTTGTTGCATTGGGATATGATAAATTGCTAGATATTGGGGATTTCCATAACTAAATATATGTTTGTTTTTCTTATGTGTTCTCTAGGTTATTACCCTGGATATGGGTCTTCTTGTTGCGGGAACAAAATATCGTGGAGAGTTTGAAGAAAGATTGAAGAAACTAATGGAGGAAATCAAACAGAGTGATGAAATAATTCTATTCATTGATGAGGTGCACACTTTAATCGGCGCAGGAGCAGCAGAAGGGGCTATTGATGCTGCTAACATCCTGAAGCCATCTCTTGCCCGAGGAGAGTTACAGGTAACCGTACAACATACATACAATTCATTTCCCTTTTACTGTTTAACGCAGATAATACCAAAAACAGAAAATGGAAAAGAAAATAAAACGTGAAACAAAGTTGGCAAAACTTGTGCGGGGTTGTACTGATTTCAGGATGGAACTATATAAACTTGTGCAGTTCCACACCCCCCCCCCCCCCTCCTTTTTAGGTTGCGCTATTGATTTAGCTTTTTCAAAAAAATCTTGAAGTTTGAGTTTCCAGTGGCCTTCTGTATTTTTTATTCAAAATAAACTAATAAATATGCTATTTTATGAACCTTTTCTTATTGATGCTGAATTAGATGATGACAAAATAGTTGAGTATGGCTTCTCAGTTATTTTACCATGTCCGTATCATTTGGAGTTCATACATCACTCTCTTTCTGAATGATTTCTACACTAGTGAATTGGATTATAGTTGATGGTTACCCTTTGAGTTATGATTCTTTGTTTTTTGGGAGCTGATTTTGGGTGAAAACTGTTTACGGAAGTGGATTGTTGGCTAATTAGCTTCAAATTATTTAATGTGCAGTGTATTGGTGCCACAACACTAGATGAATATAGAAAGCACATTGAGAAAGATCCAGCATTAGAAAGACGATTCCAGCCAGTTAAAGTGCCAGAACCGTCAGTGGATGAAACCATACAAATTTTGAAAGGGCTTCGAGAGCGATATGAAATTCACCACAAGCTTCGTTACACTGATGAAGCACTAGTTTCTGCTGCTCAGTTGTCATACCAGTACATCAGGTATGCTGTCCATGAGATACTTTGTCGTGCAGTGAGATAATTCATTTCAAGTTGGCATCTATGATCTGTTGTGTCTCATTGATAACTGAATACATATGACAATCTCTTCAACATGGAAATTCAAAGCAAAATCAAGTGTTGATATGGTAAGGGGGGTAAAAAAAAAGTATTTGGGCTTGCCCACCCCCCCTNACCTTTTTTTTTTTNAAAAAAAGTGTTGATATGGAGATGATTTTTTTTTAATCCATTTATTTTTATTTGTGTGCTTGGTAACCAAGAGAAATAGAAGAATAAAAGTGAATTTTCTGATTTTATATCATAAACTGTTTTTATTGCATTACCAATTTGGATTTTCTGCAGATGCTAACAAGACGATTTTCTGTTGTTCCTGTTTGCTTACCATGTTAATATGTGGCTGCATAACCATGTGGGATAAATAATAAATTTATTGTCCCCTGTAATGACCTGTGTTTCTGCAATCTTCTCAGTGACCGTTTTCTCCCTGATAAAGCTATTGATTTGATTGATGAAGCTGGTTCTCGGGTTCGTCTTCGTCATGCACAGGTATGAGGTGATATGGCAGTTTGTGTCTCCATCTGTCCTGTCTTGTTTCCCTTGCTTTCAATTTGATTGGTCGCTTTTTTGATACTGCAGCTCCCCGAGGAAGCTAGAGAGCTTGAAAAAGAGCTCAGGCAGATAACTAAGGAGAAGAATGAGGCTGTTCGTGGCCAAGACTTTGAAAAGGTATGAACTACCAAGTAGTGTGTGGTTGCAAGTTGGTACTTTATTTTGATACCTTGAGTCGTATCAACCGGTTGTGACACTTTTTTTCTGGTACAATTATAAATTGTTCTTTCAGAACTGAGAAATTTAATGTCTGAAAATTCAATGGTTATTTGATTGTAAAAGTTGGCATCTTGGTTCCTTTTCCAATCAAATAGATTAAGCCACCATATTATTTAGCCAACTAATGGTATATGGATAATATTTTGAACAGGCTGGAGAGTTACGTGATAGAGAAATGGACCTTAAGGCTCAGATATCTGCTCTTGTCGATAAAGGCAAAGAGATGAGCAAGGCAGAGACTGAAGCAGGGGATGTTGGTCCTGTTGTGACTGAAGTGGACATTCAACACATTGTCTCAGCTTGGACGGGCATTCCGGTTGAAAAAGTCTCGACTGATGAATCTGATCGCCTCCTCAAGATGGAAGAGACCCTTCACAAGAGAGTTATTGGTCAGGACGAAGCTGTCAAAGCTATTAGTCGTGCTATCCGGCGTGCACGTGTTGGACTCAAAAACCCCAATCGACCAATTGCTAGTTTCATCTTTTCTGGTCCAACTGGTGTCGGGAAGTCTGAACTGGCCAAAGCACTGGCTGCCTATTACTTTGGCTCAGAGGAGGCCATGATCAGGCTTGATATGAGTGAGTTCATGGAAAGACACACTGTTTCCAAGCTCATTGGTTCACCACCTGGTTATGTTGGTTACACTGAGGGTGGTCAGCTGACTGAGGCTGTTCGCCGTCGTCCTTATACTGTTGTACTCTTTGATGAGATTGAAAAGGCTCATCCTGATGTCTTCAACATGATGCTTCAAATTCTCGAGGATGGAAGATTGACAGACAGCAAGGGAAGAACTGTAGACTTCAAGAATACTCTTCTAATAATGACATCAAATGTTGGAAGCAGTGTAATTGAGAAGGGAGGACGCCGCATTGGTTTTGATCTTGATTATGATGAGAAAGACAGCAGTTACAACAGGATTAAGAGTCTGGTAACTGAGGAATTGAAGCAGTACTTCAGGCCGGAGTTCTTGAATAGATTGGATGAGATGATTGTTTTCCGACAACTCACCAAGCTGGAGGTTAAAGAGATTGCTGATATAATGCTGAAGGAGGTGTTTGATAGACTCAAGACAAAAGATATTGAGCTTCAAGTGACAGAGAGATTTAGGGAGAGGGTGGTTGAGGAAGGTTATAACCCAAGCTATGGAGCAAGGCCATTGAGAAGAGCCATTATGAGACTTTTGGAAGACAGCATGGCTGAGAAGATGCTTGCAAGAGAGATCAAAGAGGGTGATTCGGTCATTGTTGATGTCGATTCTGATGGGAACGTGACTGTGCTCAACGGCAGCAGCggtgctccagaatcattggcagatccactacctgttgtg**taa**

39B3 mutant

**atg**gctaggcttctggctcagttaactaatgcccctgctttggttcctggcggaaGACATTTCCAGTCCCAAAATGCTGGAAGATCAAGAAGGAATGTCAAAATGATGTCTTATATGCAGCCGTCTGCTTCAAGAATTAGTAGTTTCTCAGGATTGCGACGCTCCAATGCTTTAGATACATTCACCTGTGATTTCCATTCCACGGTGGCTGTTTCACTCTCTTCTAGACGAGGAAAGTCTGGAAGAGCTGGTGGATTTGTTGTTAAAGCCATGTTTGAGCGCTTCACAGAGAAAGCAATTAAAGTGATTATGCTTGCCCAAGAGGAAGCAAGACGGCTTGGCCACAATTTTGTTGGCACGGAGCAAATTCTATTGGGTCTTATTGGTGAAGGCACTGGCATTGCTGCTAAGGTTCTTAAATCAATGGGAATCAATCTTAAAGATGCACGTGTAGAAGTGGAGAAGATTATTGGAAGGGGAAGTGGTTTTGTTGCTGTGGAAATTCCGTTCACTCCTCGTGCAAAGCGTGTCTTGGAGCTTTCTCTGGAGGAAGCCCGCCAGCTGGGTATCATAACCTTCTCCTTTCCAGTTTAGCAATGCCTTATGTTTTAGGAACTAGGATCTTTTTGGATTTCACTATAGGATTACTGTTTTAAATTCTGTTGGCCTGTCTCTTAACTGCGTGATTGAGCGTATCAGGTGCTTTAATTTTTGATATATTATCCGTTGGAACCTTTGCTTCGGGTCATTTGTTATTGGCCATATTTTGGGCTTAATCCTTCTACATATGTATCACTTTGATTTCCAAGTTCTTGAAGCCAAATTCTCTCAAATTTCAATCTCAGAAACTCACAAAAGTTTATATATGTGATGAAATGTTAAAAAATCATAGAGAAAAGTTGTTTAAGTTGATTCTTTAACCTTGAATTAATTATCTAATTTGTTATTGACTTTATTAGATCTAGAATTGGTTTTCCATTTGATTCTACTGAACTAAAGAAAATATAAATTAAAGCAATTTTTTTTTTGTTACATTGTTACTTTTGTAGCATCCAATTCATATGACCTTTGTTTCTGATTTTTAAAAGCAAGTGTTAGCTTCTTGAAATATATTATTTTCATTTTCTGACTAGGAAACACATCTAATTGTCATCTGAAAAATGTATACTTACTTGAAATTTCAGGCCATAATTATATTGGGTCCGAGCATCTTCTCCTTGGGCTTCTTCGTGAAGGGGAAGGTGTAGCAGCTCGTGTTCTTGAGAATTTAGGTGCTGACCCTAGTAACATCCGCACACAGGCAAGCAACAGTTCTTATCATTTGTGTTCCGATCTGATTTTCTTTCCTTGAGACCTGTTTCATTGCTAGCTTTGTTAAATAAAAAATTGATTTGATATTCTTAGTAAAAGATATAAATAGGCAAAGCTTTTGTTGGAGTGAACAGGTTATTCGTATGGTTGGTGAGAGCACAGAAGCTGTTGGCGCTGGTGTTGGAGGAGGAAGCAGTGGCAACAAGATGCCAACACTGGAGGAGTATGGAACAAATTTGACTAAGCTAGCAGAGGAGGTAATATGCGGTGACGTACATTGCAAAATTTGCTACTTAGTTTTTGAGGCATTTTTCTAAGTTTAGTTTCTTCTCGTTTCAGGGAAAATTGGATCCTGTTGTTGGGAGACAGCCACAAATAGAGCGTGTTGTCCAAATTTTGGGCCGTCGAACTAAGAATAATCCATGTCTTATTGGAGAACCTGGTGTGGGTAAAACAGCAATTGCAGAAGGCCTTGCTCAACGGATTGCAAGTGGTGATGTTCCTGATACCATTGAAGGAAAGAAGGTTGGTATAATCAGATATAGTTGTTAGCTATTTAACATGGGTACATTAACATTTTGAATCTTATAATCGAGGATGAAAGAACTTCCTTACCTCGTTTTTGAAGAATTTAATAGGAGCATTTGCCAACTTTGAGCTGTACTGGTCTGTATGTATATATGTGAACTCTTTGCATTAAAAAAGTTGCTTTTTCTTTTTTTGCATTTTTTTAACTAATTGAAAGTTATACCATAAGCTCTCTCTCTGAGATTTAAAGATGGCTGGTTAAGTGTCATTCAGAAGCTTTTGAATAATAGTCAGTCGTCTACTTGACATGGATACTTCATTTATTCCGAAGGCCAATCTTTCATATGACAGCTTGTGGATCTGGTGATGGGAATTTGATTTTTTTTTGTCATTTAAAATTTTGCATTTATTACTTGGAAAAATGTGTATCGTTAGTGTGAATTAAAAAAAAAAAACTGTTCGTATCATAAATGAATATATAATTAGGCATGTTGCACAAGAAGATGATATGAAGCATTTTATTTCAACTTCAATTCTGTTTGCAGCTTTACATATTTATGTAGTTATATTGATTTGGTGTGTGAACATTTAAATGATATGAAAAACCTTTTGAGTTAATTTAGATGTGTACTCATGTCATGTAGATGTTTTTTCTGCACAATATATTTGTCGATGGATTCGACTCTTGTTCTAAATATTTGATATTCATTTTGAAGAGAGAATACTTTATCTGCTGGTGATTGCTTGATTTGTATTCAAATGCACCAGAGTTATTGGATGAATGTAACCATGTGTATCCTGTAAAAAAGATGTAAGATTTCTGCTGCAAAACTTTAAACTGGCCTTTGACGCTTCTTCATTAGGTGCAGCGTGAATTTATAATTTCTCATAGAATGCTGTAACCTGAGGCTAGGTGGTTGCCACTAAATGAAGGATGTGGTGACCATTTATCATAATTTTATGTCCTGAAATTCAAATTTTTACACTGTTTGATGCTCTGGTTGTTGCATTGGGATATGATAAATTGCTAGATATTGGGGATTTCCATAACTAAATATATGTTTGTTTTTCTTATGTGTTCTCTAGGTTATTACCCTGGATATGGGTCTTCTTGTTGCGGGAACAAAATATCGTGGAGAGTTTGAAGAAAGATTGAAGAAACTAATGGAGGAAATCAAACAGAGTGATGAAATAATTCTATTCATTGATGAGGTGCACACTTTAATCGGCGCAGGAGCAGCAGAAGGGGCTATTGATGCTGCTAACATCCTGAAGCCATCTCTTGCCCGAGGAGAGTTACAGGTAACCGTACAACATACATACAATTCATTTCCCTTTTACTGTTTAACGCAGATAATACCAAAAACAGAAAATGGAAAAGAAAATAAAACGTGAAACAAAGTTGGCAAAACTTGTGCGGGGTTGTACTGATTTCAGGATGGAACTATATAAACTTGTGCAGTTCCACACCCCCCCCCCCCCCTCCTTTTTAGGTTGCGCTATTGATTTAGCTTTTTCAAAAAAATCTTGAAGTTTGAGTTTCCAGTGGCCTTCTGTATTTTTTATTCAAAATAAACTAATAAATATGCTATTTTATGAACCTTTTCTTATTGATGCTGAATTAGATGATGACAAAATAGTTGAGTATGGCTTCTCAGTTATTTTACCATGTCCGTATCATTTGGAGTTCATACATCACTCTCTTTCTGAATGATTTCTACACTAGTGAATTGGATTATAGTTGATGGTTACCCTTTGAGTTATGATTCTTTGTTTTTTGGGAGCTGATTTTGGGTGAAAACTGTTTACGGAAGTGGATTGTTGGCTAATTAGCTTCAAATTATTTAATGTGCAGTGTATTGGTGCCACAACACTAGATGAATATAGAAAGCACATTGAGAAAGATCCAGCATTAGAAAGACGATTCCAGCCAGTTAAAGTGCCAGAACCGTCAGTGGATGAAACCATACAAATTTTGAAAGGGCTTCGAGAGCGATATGAAATTCACCACAAGCTTCGTTACACTGATGAAGCACTAGTTTCTGCTGCTCAGTTGTCATACCAGTACATCAGGTATGCTGTCCATGAGATACTTTGTCGTGCAGTGAGATAATTCATTTCAAGTTGGCATCTATGATCTGTTGTGTCTCATTGATAACTGAATACATATGACAATCTCTTCAACATGGAAATTCAAAGCAAAATCAAGTGTTGATATGGTAAGGGGGGTAAAAAAAAAGTATTTGGGCTTGCCCACCCCCCCTGACCTTTTTTTTTTTAAAAAAAAGTGTTGATATGGAGATGATTTTTTTTTAATCCATTTATTTTTATTTGTGTGCTTGGTAACCAAGAGAAATAGAAGAATAAAAGTGAATTTTCTGATTTTATATCATAAACTGTTTTTATTGCATTACCAATTTGGATTTTCTGCAGATGCTAACAAGACGATTTTCTGTTGTTCCTGTTTGCTTACCATGTTAATATGTGGCTGCATAACCATGTGGGATAAATAATAAATTTATTGTCCCCTGTAATGACCTGTGTTTCTGCAATCTTCTCAGTGACCGTTTTCTCCCTGATAAAGCTATTGATTTGATTGATGAAGCTGGTTCTCGGGTTCGTCTTCGTCATGCACAGGTATGAGGTGATATGGCAGTTTGTGTCTCCATCTGTCCTGTCTTGTTTCCCTTGCTTTCAATTTGATTGGTCGCTTTTTTGATACTGCAGCTCCCCGAGGAAGCTAGAGAGCTTGAAAAAGAGCTCAGGCAGATAACTAAGGAGAAGAATGAGGCTGTTCGTGGCCAAGACTTTGAAAAGGTATGAACTACCAAGTAGTGTGTGGTTGCAAGTTGGTACTTTATTTTGATACCTTGAGTCGTATCAACCGGTTGTGACACTTTTTTTCTGGTACAATTATAAATTGTTCTTTCAGAACTGAGAAATTTAATGTCTGAAAATTCAATGGTTATTTGATTGTAAAAGTTGGCATCTTGGTTCCTTTTCCAATCAAATAGATTAAGCCACCATATTATTTAGCCAACTAATGGTATATGGATAATATTTTGAACAGGCTGGAGAGTTACGTGATAGAGAAATGGACCTTAAGGCTCAGATATCTGCTCTTGTCGATAAAGGCAAAGAGATGAGCAAGGCAGAGACTGAAGCAGGGGATGTTGGTCCTGTTGTGACTGAAGTGGACATTCAACACATTGTCTCAGCTTGGACGGGCATTCCGGTTGAAAAAGTCTCGACTGATGAATCTGATCGCCTCCTCAAGATGGAAGAGACCCTTCACAAGAGAGTTATTGGTCAGGACGAAGCTGTCAAAGCTATTAGTCGTGCTATCCGGCGTGCACGTGTTGGACTCAAAAACCCCAATCGACCAATTGCTAGTTTCATCTTTTCTGGTCCAACTGGTGTCGGGAAGTCTGAACTGGCCAAAGCACTGGCTGCCTATTACTTTGGCTCAGAGGAGGCCATGATCAGGCTTGATATGAGTGAGTTCATGGAAAGACACACTGTTTCCAAGCTCATTGGTTCACCACCTGGTTATGTTGGTTACACTGAGGGTGGTCAGCTGACTGAGGCTGTTCGCCGTCGTCCTTATACTGTTGTACTCTTTGATGAGATTGAAAAGGCTCATCCTGATGTCTTCAACATGATGCTTCAAATTCTCGAGGATGGAAGATTGACAGACAGCAAGGGAAGAACTGTAGACTTCAAGAATACTCTTCTAATAATGACATCAAATGTTGGAAGCAGTGTAATTGAGAAGGGAGGACGCCGCATTGGTTTTGATCTTGATTATGATGAGAAAGACAGCAGTTACAACAGGATTAAGAGTCTGGTAACTGAGGAATTGAAGCAGTACTTCAGGCCGGAGTTCTTGAATAGATTGGATGAGATGATTGTTTTCCGACAACTCACCAAGCTGGAGGTTAAAGAGATTGCTGATATAATGCTGAAGGAGGTGTTTGATAGACTCAAGACAAAAGATATTGAGCTTCAAGTGACAGAGAGATTTAGGGAGAGGGTGGTTGAGGAAGGTTATAACCCAAGCTATGGAGCAAGGCCATTGAGAAGAGCCATTATGAGACTTTTGGAAGACAGCATGGCTGAGAAGATGCTTGCAAGAGAGATCAAAGAGGGTGATTCGGTCATTGTTGATGTCGATTCTGATGGGAACGTGACTGTGCTCAACGGCAGCAGCggtgctccagaatcattggcagatccactacctgttgtg**taa**
